# Social prescribing for people with complex needs: a realist evaluation

**DOI:** 10.1186/s12875-021-01407-x

**Published:** 2021-03-18

**Authors:** Emily Wood, Sally Ohlsen, Sarah-Jane Fenton, Janice Connell, Scott Weich

**Affiliations:** 1 School of Health and Related Research, The University of Sheffield, Regent Court, 30 Regent Street, Sheffield, S1 4DA UK; 2 Institute for Mental Health, School of Social Policy, University of Birmingham, Edgbaston, Birmingham, B15 2TT UK

**Keywords:** Social Prescribing, Salutogenesis, Realist evaluation, Co-morbidity, Multi-morbidity, Depression

## Abstract

**Background:**

Social Prescribing is increasingly popular, and several evaluations have shown positive results. However, Social Prescribing is an umbrella term that covers many different interventions. We aimed to test, develop and refine a programme theory explaining the underlying mechanisms operating in Social Prescribing to better enhance its effectiveness by allowing it to be targeted to those who will benefit most, when they will benefit most.

**Methods:**

We conducted a realist evaluation of a large Social Prescribing organisation in the North of England. Thirty-five interviews were conducted with stakeholders (clients attending Social Prescribing, Social Prescribing staff and general practice staff). Through an iterative process of analysis, a series of context-mechanism-outcome configurations were developed, refined and retested at a workshop of 15 stakeholders. The initial programme theory was refined, retested and ‘applied’ to wider theory.

**Results:**

Social Prescribing in this organisation was found to be only superficially similar to collaborative care. A complex web of contexts, mechanisms and outcomes for its clients are described. Key elements influencing outcomes described by stakeholders included social isolation and wider determinants of health; poor interagency communication for people with multiple needs. Successful Social Prescribing requires a non-stigmatising environment and person-centred care, and shares many features described by the asset-based theory of Salutogenesis.

**Conclusions:**

The Social Prescribing model studied is holistic and person-centred and as such enables those with a weak sense of coherence to strengthen this, access resistance resources, and move in a health promoting or salutogenic direction.

## Background

The popularity and prominence of social prescribing (SP) is growing. In October 2018, the UK Government committed to investment in SP as part of its Loneliness Strategy [1]. The National Health Service Long Term Plan [2] also highlights a key role for SP in the provision of health and social care in the UK. SP is not one intervention, it is a pathway [3]; it is an umbrella term that encompasses interventions that are asset-based, person-centred and typically involve a named professional who supports the person and helps link services and agencies involved in their care [4, 5]. Asset-based approaches seek to positively to mobilise the assets, capacities or resources available to individuals and communities which enable them to gain control over their lives and circumstances [6]. NHS England define personalised care (or person-centred care) as people having choice and control over the way their care is planned and delivered based on what matters to them and their individual strengths and needs [7]. Most SP interventions aim to support individuals to have greater control over their own health [8], for instance through exercise or benefit advice programmes [9]. While evidence for social prescribing is broadly supportive [10], rigorous studies of effectiveness and cost-effectiveness remains scarce [9, 11]. Previous reviews found indications that SP may be effective in improving health and well-being and reducing healthcare usage but included studies were small and many had low methodological quality, reducing confidence in the outcomes [9, 11] meaning the existing evidence base for SP as a means of improving health outcomes is poor. By contrast, there is considerable support for SP at policy and commissioning levels.

The link worker role in SP is considered key for people with multiple long-term health conditions where care can often be fragmented [7]. This resonates with collaborative care models [12] which have four elements [13]: multi-professional approach (with one acting as a case coordinator); enhanced communication between professionals; structured management plan; and scheduled follow up. We therefore hypothesised that the underlying mechanism for SP might be consistent with collaborative care [14].

This research set out to elucidate the mechanisms that facilitate engagement and positive outcomes with SP intervention among people with multiple health conditions and social needs. We sought to develop, test and refine the initial programme theory (that the benefits associated with SP derive from enacting collaborative care) using stakeholder experiences.

## Methods

### Realist evaluation

Realist evaluation (RE) looks at generative causation [15], a key strength is modelling complexity. Adopting a realist approach enabled us to go beyond identifying ‘barriers’ and ‘facilitators’ [16] to uncover underlying mechanisms and understand how, when and why they are activated. RE requires researchers to have an initial theory that can be challenged and refined during the study. Mid-range theories (which may or may not be novel) can be applied to the findings to improve generalisability of case study findings [17, 18].

RE recognises that wider context influences outcomes and specifically how individual actors respond to different parts of the intervention at different times. To describe this complexity, statements describing pathways between intervention or individual Context (C), and underlying Mechanisms (M) that subsequently shape patterns of outcomes (O) are created (CMO configurations) [15]. CMO configurations aim to describe why a person (or case) responds to an intervention in a certain way and how this can change depending on circumstances. CMOs can be linear or more complex [15, 19].

We focused on one large voluntary sector, community anchor organisation providing SP to an inner-city area of high socioeconomic deprivation, predominantly deprived white working class council estates. Clients often have multiple health conditions including co-existing physical and mental health concerns. Most have social needs associated with housing, benefits and lack of support networks. Many are isolated. Unlike some areas, there was no central referral point; general practices and other referrers (such as housing officers) referred to one or more of many community anchor organisations that provide services in their local area (Table 1 provides details of the setting). In some parts of the country, general practices have begun employing link workers but in this area, all social prescribing is provided by these third sector organisations, link workers are not provided by primary care networks.

**Table 1:** Details of the study setting

**Organisation Type:** Community Anchor Organisation (Voluntary sector)
**Location:** Inner-city area of high socioeconomic deprivation in a city in the north of England
**Referral type:** General Practitioners and other services refer direct to the organisation. Self-referral is also possible. Triage worker signposts to the most appropriate service, based on the client’s personal goals rather than the doctor’s determination of the problem. In 2018, there was a total of 1372 referrals; 813 from General Practitioners, 207 self-referrals, 315 from other sources (e.g. housing, community mental health teams) and 37 whose referral status was not recorded. The number of clients with a mental health condition is not recorded; however, in 2018, 56 clients enrolled on the coping and self-management programme and 59 enrolled on the emotional well-being programme
**Services provided:** Advice and services around health, employment and training. For the purposes of this study, we only considered the health section: this includes health training (e.g. weight loss or health eating advice, alcohol or cigarette reduction and exercise advice), social café’s, benefits and housing advice, and volunteer work. There is no set pathway through the service. Clients can access different services at different times in whatever order meets their needs. The service has no time limit
**Staff team:** Paid health trainers, health activity workers and advocacy workers (primarily giving benefits and housing advice) and unpaid volunteers. Any of the paid workers could be a link worker, this would be decided based on client goals. Clients with predominantly health goals would have a health trainer as a link worker. Once these goals were met, the client may be referred to other colleagues if needed, for example for benefits advice. The person acting as link worker would change
**Care pathway:** New clients are screened by the in-house triage service to ensure the client is seen by the right part of the service to meet their goals. It is possible to move from one service to another or see multiple workers at one time depending on the nature of the client’s personal goals. The service also includes social café’s, which can take referrals or clients can drop in. You can attend the social café concurrently with other one to one services

**Fig. 1:**
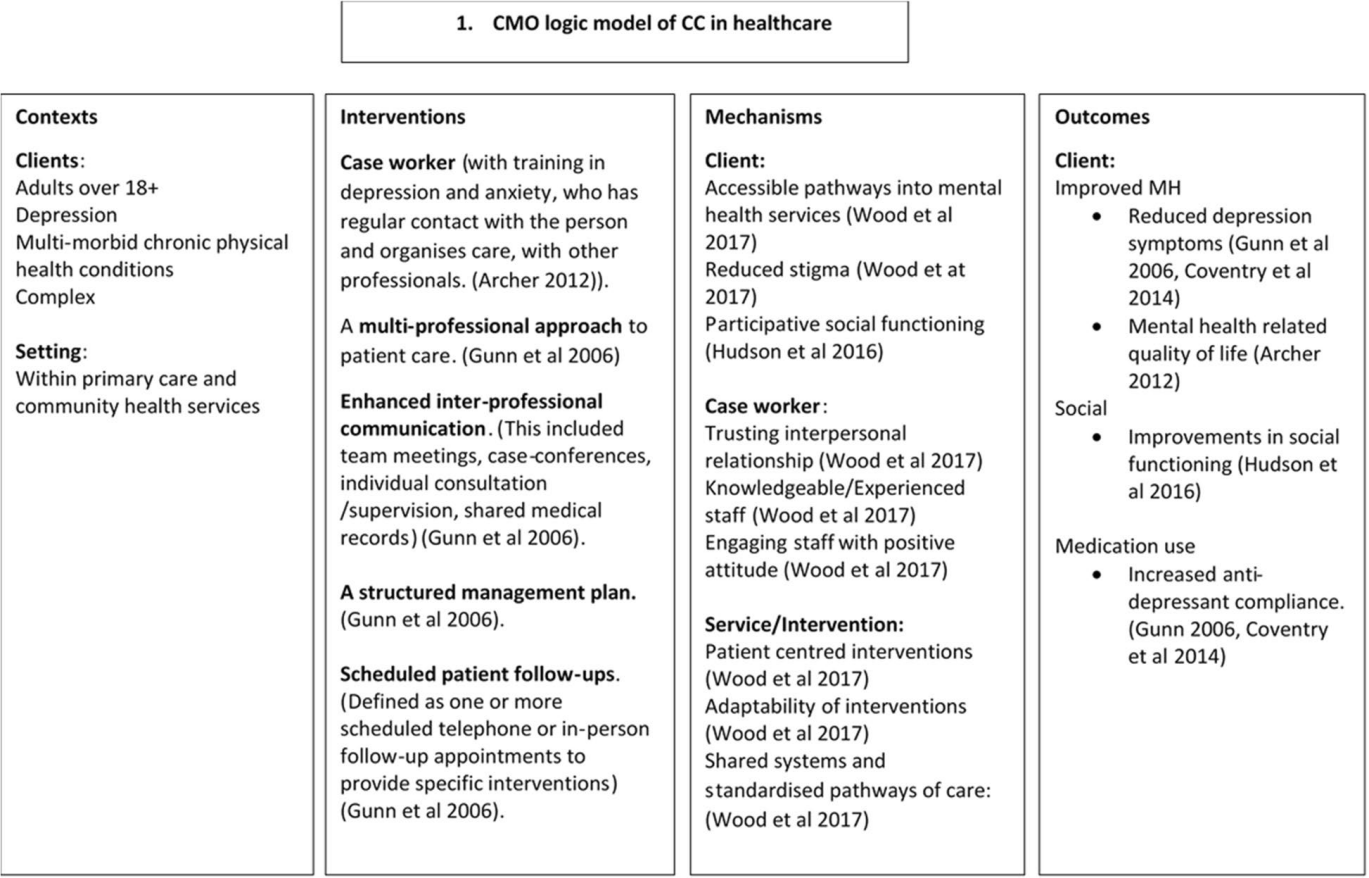
The initial programme theory

The initial programme theory (IPT) was informed by the wider research team and relevant literature reviews of collaborative care [20], realist methods [18] and social prescribing [9, 10, 21]. It was formed over multiple meetings between researchers and stakeholders from the host site as well as another SP organisation in the same city, to further understand why and how SP works for certain populations. The IPT to be tested was that collaborative care and SP are overlapping concepts (Fig. 1). We hypothesised that SP organisations are potentially effective in improving self-management for adults with co-existing physical and mental health conditions because they work on a collaborative care model [12]. For the purpose of this study, intervention type was disaggregated from context in order to differentiate between interventions in one contextual setting.

The study was iterative in nature and conducted over three phases. Stakeholders from the host community anchor organisation were included in development and design meetings from the start. This helped ensure that the research was appropriate for the local model of SP delivery and that it would provide insights of value to both community anchor organisation s and researchers. Phase one tested the IPT through data collected in interviews with people working for the organisation, those receiving SP and external referring organisations, to test and refine this theory. In Phase two, we developed CMO statements which refuted the IPT, leading to modification of the theory. Phase three focused on applying and developing wider theory to synthesise the CMOs into a mid-range theory. A mid-range theory is a theory that lies between working hypotheses, contains testable predictions and evolves as efforts are made to develop a unified theory. It was developed and related, where appropriate, to existing theories [17, 18]. The mid-range theory provides a level of abstraction to the analysis and therefore a generalisability beyond the immediate context [17].

### Data collection

#### Phase 1: interviews

We aimed to interview stakeholders with different points of view and experiences of SP. Clients were approached if they met inclusion criteria of having both physical and mental health issues. Recruitment was via convenience sampling and took place at different services within the organisation: health training, advocacy, volunteer development and social cafes. At the social cafes one of the researchers gave a short talk to attendees to introduce the research and who we were looking for as participants. Attendees were then asked to approach either the researcher or café staff if they wished to participate. For all other services, the staff member suggested the research to their client on a one to one basis. Everyone was told participation was optional.

All staff from the host SP organisation were invited to interview via emails detailing the research. Doctors and practice nurses at local general practices were invited to interview as SP referrers. All practices associated with the host organisation were approached.

The interview schedule evolved via a constant comparative method [22] such that each interview was informed by the ones that had taken place before it. The focus of the interviews was to present components of the IPT to the interviewee, on flash cards (face-to-face interviews for staff and clients) or verbal hypothesis (telephone interviews for referrers) for them to comment on with a view to providing theory refinement [23]. The interview structure and questions were adapted from the RAMESES-II project [24] and flash cards were adapted according to who was being interviewed.

Interviews were audio recorded and lasted between 15 and 60 min. Participants signed a consent form before the interview commenced. Most interviews took place face-to-face in the host organisation’s premises, though referrer interviews were conducted by telephone as this was more convenient for clinicians. Interviews were conducted by EW and SO between February and April 2018.

#### Phase 2: stakeholder workshop

A workshop with key stakeholders [18] supported refinement of the coding frame and analysis of emerging themes. The results contributed to the emerging mid-range theory and CMO configurations.

Twenty participants were invited to the workshop. They were referrers into the service (e.g. National Health Service staff), SP staff, or clients. Referrers and clients were only invited if they had not been previously interviewed to get a wider range of opinions. As before, clients self-identified as having co-morbid physical and mental health issues. Referrers came from different sectors including primary care, housing, and community mental health teams and had referred at least one client to the service. As we had interviewed almost all the host SP staff members, most staff members at the workshop had participated in interviews.

Initial results from the interviews were reported at the workshop, to gauge attendees’ views on our interpretations and areas where we considered there may be missing data. Participants were asked to provide comments, criticisms and feedback on those interpretations. These were then incorporated into final analyses.

The workshop lasted for three hours and clients received a store voucher for attending.

### Analysis

Realist analysis takes an iterative approach, moving between different sources of data and using deductive and inductive reasoning [25] (see Table 2).

**Table 2:** Key elements of the Realist analysis process

**The realist methodology uses the following approaches judiciously and in combination:**
• Organizing and collating primary data and producing preliminary thematic summaries of these
• Repeated writing and rewriting of fragments of the case study
• Presenting, defending, and negotiating particular interpretations of actions and events both within the research team and also to the stakeholders themselves
• Testing these interpretations by explicitly seeking disconfirming or contradictory data
• Considering other interpretations that might account for the same findings
• Using cross-case comparisons to determine how the same or submechanism plays out in different contexts and produces different outcomes, thereby allowing inferences about the generative causality of different contexts
From Greenhalgh, T., Humphrey, C., MacFarlane, F., Bulter, C., & Pawson, R. (2009)

The IPT was interrogated using data collected at interview, by testing our hypothesis that SP is consistent with collaborative care [26]. This was done by analysing experiences of three stakeholder subgroups (clients, staff and referrers) to see if their views corroborated or differed from the IPT. A deductive approach was used. Analysis consisted of applying data to the four key elements of collaborative care using a Framework approach. Themes about what worked for whom, when and why that did and did not fit with the IPT were used to continually refine the model. Coding was undertaken by SO; EW and JC independently coded 14 interviews to cross check the coding frame. NVivo software was used to assist in data management.

The developing themes became the premise for disputing the IPT as the data were related to wellbeing not ‘health’ and ‘disease management’. These were developed into a series of CMO configurations by the wider research team until agreement was reached. New configurations were then tested with data collected from the workshops in phase two.

Finally, we looked to extend generalisability beyond the immediate context [17] by relating, where appropriate, the newly created theory (mid-range theory) to existing theories [17, 18]. Client-led interventions and asset-based approaches emerged as key to the mid-range theory. This is closely linked with salutogenesis [27, 28].

Salutogenesis focuses on promoting health and well-being as opposed to managing symptoms of disease. It further posits that life experiences shape one’s sense of coherence (ability to comprehend a situation, find meaning and be able to act). A strong sense of coherence aids in mobilising resources (internal and external) for dealing with stress and helping a person move towards health rather than disease [27, 29]. Salutogenic interventions are those which seek to strengthen a person’s ‘sense of coherence’ [29].

## Results

### Participants

The realist analysis was based on 35 interviews with clients (*n* = 15), staff (*n* = 13), and referrers (*n* = 7) who were all involved the health section of the SP community anchor organisation. All but one member of host SP staff (who was on leave during recruitment) from the health section were interviewed in phase one. Staff backgrounds varied and included health trainers, benefits/housing advisors and people who supported the organisation’s volunteers. The health section manager was also interviewed.

The stakeholder workshop had 15 delegates including seven staff, five referrers and three clients. Five staff at the workshop had been involved in the interviews. Two senior managers attended who had not been invited to interview. None of the referrers or clients at the workshop had been interviewed in a deliberate attempt to ensure we were getting a range of viewpoints. See Table 3 for participant information.

**Table 3:** Demographic details of the participants

**Interviews**	
Referrers	*N* = 6 (general practitioners, practice nurse, 2 male, 4 female)
Staff	*N* = 13 (1 manager, 4 health trainers, 3 advisors, 3 volunteer coordinators 2 triage, 6 males, 6 females, one preferred not to say)
Clients	*N* = 15 (12 clients, 3 clients who also volunteer, 5 males, 9 females, 1 preferred not to say)
**Focus group**	
Referrers	*N* = 5 (community mental health team, housing, social work)
Staff	*N* = 7 (2 managers, health trainers, advisors)
Clients	*N* = 3 (2 male and 1 female)

### Modification of the IPT

**Fig. 2:**
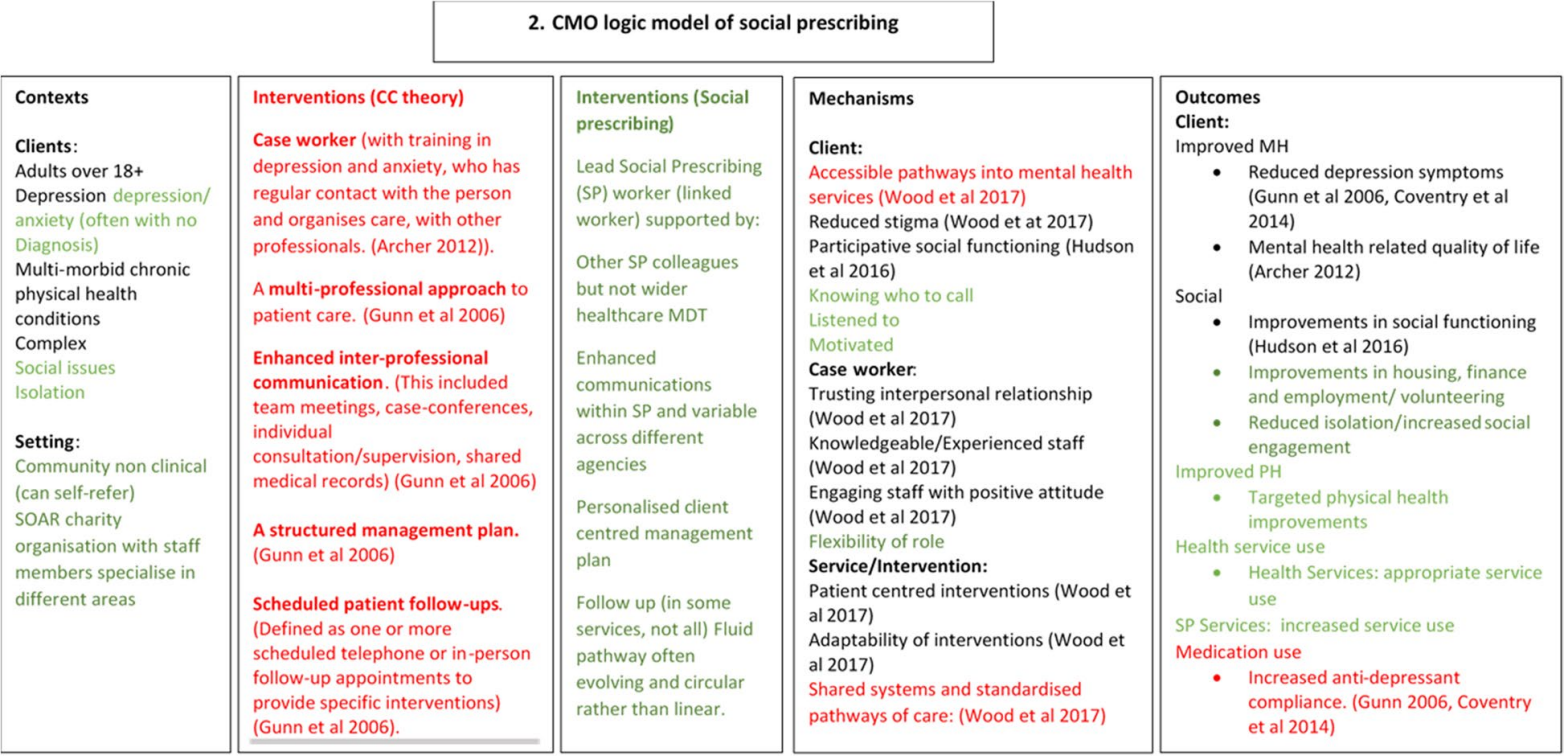
The mid-range theory developed after interviews with SP stakeholders

The IPT was that SP works in a similar way to collaborative care. We asked participants about specific elements of their experiences that would have aligned with collaborative care had it been a key underlying mechanism. They were then asked about what they felt was helpful about attending the SP service. When researching whether an intervention ‘works’ it is important to define its aims. One of the aims of SP is to improve health and well-being. However, other outcomes necessarily exist, the service is client-led so clients will have their own desired outcomes, which may or may not link directly to improved health as defined by health services. The answers allowed us to refine the IPT (Fig. 2).

The interventions in SP (Fig. 2), although superficially similar to collaborative care, were different in practice and activated a wider range of mechanisms and outcomes.

**Fig. 3:**
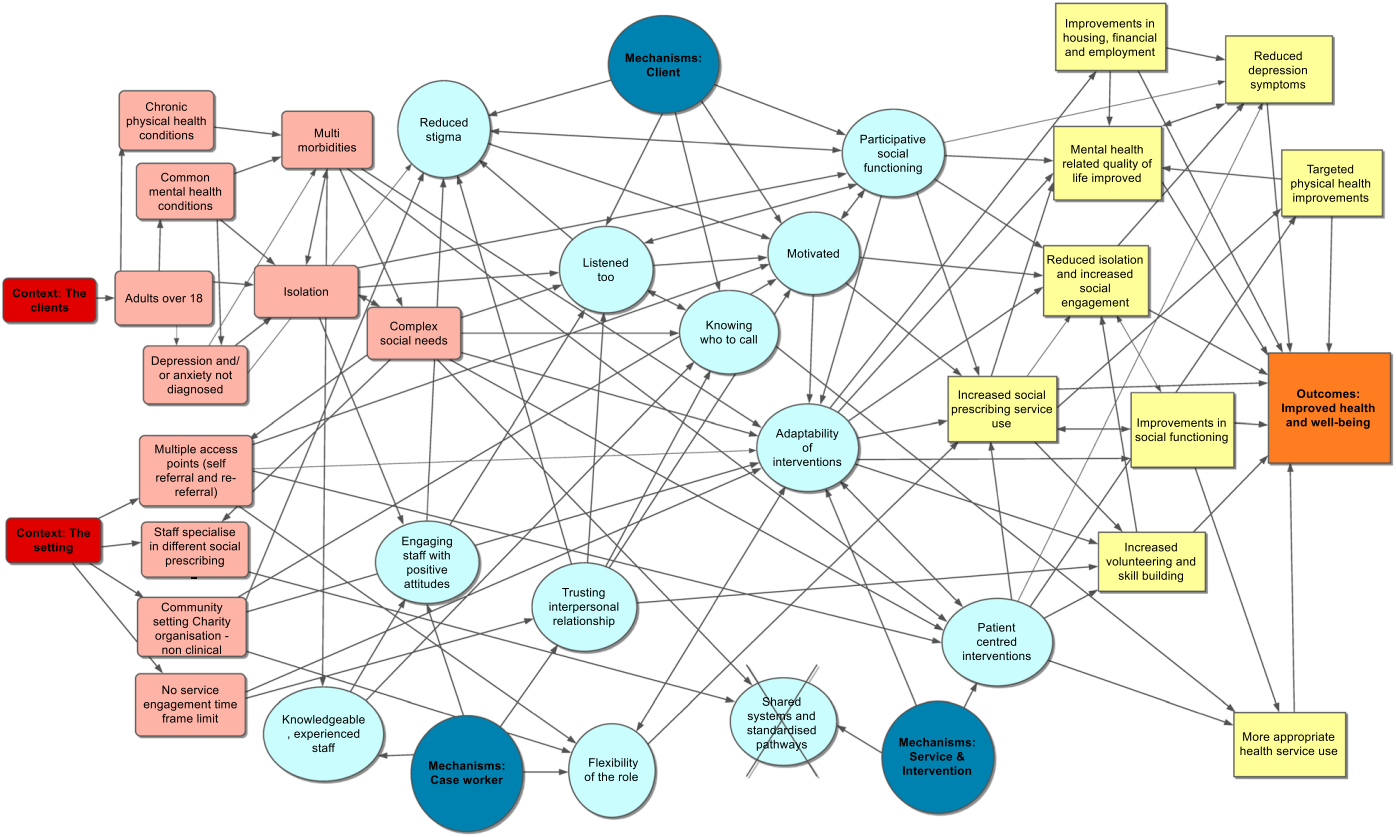
Diagram detailing some of the main CMOs and their interlinking nature

The CMO configurations derived from the data resulted in multiple interconnections, especially between mechanisms and outcomes, which did not fit [19] simple linear progressions such as ‘C + M = O’ [15]. Many configurations reported multiple contexts resulting in multiple mechanisms, which lead to an outcome attained which then became a mechanism to achieve another outcome. For example, many clients were referred or self-referred to the organisation because they were isolated (context). The social cafes (intervention) facilitated making new social connections (mechanism) which reduced isolation (outcome). Although this follows the CMO framework it does not represent the complexity found in the data, which were more multifaceted than the above statement implies. The familiar location of the cafés, which are set in local communities, was also a mechanism for increased engagement, and therefore also reduced isolation. As did the increased confidence (Mechanism (M)) clients gained from trusting staff (M) who could work flexibly (M) and were not sited within the NHS (Context (C)) but were still knowledgeable (M). Intermediate outcomes can, in turn become mechanisms for longer term outcomes. For example, increased engagement could be an outcome in its own right but it is also a mechanism for improved mental and physical health. Figure 3 shows an overview of the interweaving CMOs that we discovered.

Some of the configurations demonstrate the overlaps and differences between the original and modified IPTs. Points marked with a * are from the modified IPT but not the original, unmarked points stem from both, supporting quotes are illustrated below.

### Non-stigmatising environments

Adults with depression but no diagnosis/treatment* (C) are accessing SP services in community settings* (C), this is perceived as less stigmatising (M) and intimidating than clinical services* (M), resulting in increased engagement (Outcome (O)) and improved mental health (O).

*SP [organisations] are there for people with low confidence (C) so they’re not going to look down on you (M). Client 1*

*Chatting to people (M), you know you’re not on your own (M). You know you’re not the only person who’s had problems (C/M). Client 3*

This SP organisation offers a welcoming setting that helps the client’s mental health but does not focus on it directly.

Adults who are isolated* (C) possibly as a result of a bereavement* (C) with mental health issues (C) are receiving personalised client centred management plans in SP* (Intervention). This is can be provided on a flexible basis to meet their need* (M), partly due to the flexibility of the staff roles (M) and staff having time to listen* (M); resulting in improved Mental Health (O) and more appropriate health service use* (O).

Staff felt clients valued this approach as they would receive word of mouth self-referrals from friend and family of existing clients.

### Person-centred care

When thinking about person centred interventions one staff member spontaneously described their own idea of the CMO configuration.

*When someone is new to the service (C), it’s crucial that we do it in an approached manner (M) that we can do it at their speed, (M) feeling comfortable about it (O), giving confidence that they can do it (M), and allowing that to flourish (O) and say ‘come on, we can move forward’ (M). So, it’s empowered them (O), they’ve got to make that choice (M) and they’ve got to make those decisions but it’s about being supportive isn’t it (M), to doing it. And that’s what I see my role, is supporting people and moving them on to next… every individual has structured management plan (O), speaks for itself. Every one’s different. It’s not my plan. It’s their plan (M). Staff 11*

This suggests that staff valued the flexibility of their role working at the client’s pace, seeing it as integral to offering person-centred care. ‘It’s not my plan, it’s their plan’ implies that their role is to facilitate and guide the individual to choose rather than ‘intervening’ in the classic model. Whilst many health care workers do have a degree of autonomy and flexibility, they are usually constrained in this. The SP workers here described a level of working—supported by management—to meet the needs of clients however unusual, for example, aiding in house clearance for a hoarder.

### Social isolation

Clients with the similar contexts attend social cafes: Have a space/location to engage in participative social function (meet people/peers/friends/shared experiences)* (M) are listened to (by peers)* (M), which improves/increases social functioning (O) and reduces isolation* (O) and improves mental health (O).

*It’s building my confidence up great (C/O). I’m making loads of friends (O). I mean, I’m in a craft group but I don’t really do much crafting when I’m It’s more chatting (M) and helping the others (M), so it’s lovely, and they’re just so friendly(M). Client 14*

*Instead of once a fortnight, they’re going to somewhere twice a week now (O) so there’s, there’s always something for them to do (M) and it brings them together (M), I mean they say to me, things like oh, if I didn’t come here I’d have nowhere else to go (O), I’d be sat, four walls (C), I don’t know what I’d do if I didn’t have this group, and that type of thing. Staff 7*

Clients value the peer support that the social cafes’ provide; reducing not only physical isolation but also emotional isolation by introducing clients to people with similar issues, who are able to support each other. This reduction in isolation was also felt to have an effect on the physical health of clients and ultimately their attendance at General practice clinics. For elderly widowed males who had been dependent on their partner for essential life skills (cooking) (C) personal help with shopping and guidance may increase confidence and motivation (M) to eat better and lose weight (O).

By regularly attending SP activities(C), clients often meet others who have been through similar situations (C) it creates a social network(O/M), shared experience gives peer support (O), Reducing sense of isolation and reliance on health services being the only place you can discuss your health(M/O). Leading to reduced primary health care use. (O).

### Wider determinants of health

Wider determinants of health are a range of economic, social and environmental factors that directly and indirectly affect people’s health [30]. Clients present with social issues* (C) contributing to mental health issues (C), staff in SP have skills to support social issues* (C). Clients receive personalised management plans, the interventions are adaptable to this individual need* (M) with staff being flexible in how this support is offered/delivered* (M) Resulting in improvements in housing/finances/employment* (O) and positive impacts on mental health (O).

*We can even do a home visit (Intervention*), because even asking someone to come and see us here for the first time is daunting (C). so I think with us, slotting in with them (M), I see it as like a jigsaw, so it just all slots in because they see us and we look at the barriers to health and put them steps in first and work through them with them (M), and then it’s just giving them that bit of self-belief that they can do something and show them how they can make small changes that that’s leads to bigger things so by us being there, they can then move on to volunteering (O) and then move on to employment advocacy (O), if they are on *[employment and support allowance] they can help them sort out the benefits and what have you but then they can refer back into us again, to say well actually they are on ESA but they are looking at wanting to return to work but they have got no confidence (C), you know, so we sort of can keep seeing them (M). Staff 9*

SP in this organisation contributed to health improvements by concerning themselves with the wider determinants of health, not just presenting health concerns.

### Poor interagency communication

Clients present with complex social issues* (C). Clients receive personalised management plans (Intervention). But poor shared systems with external organisations (M) and external organisations perceiving SP staff as non-professional (M) resulted in difficulty with systematic sharing of information (O) and longer waits with more chance for clients to disengage (O).

*I think that would make a huge difference, because if a [General Practitioner] was to log in and see that they're working with social prescribers and they're going to groups and this has happened and that has happened, then we can work and keep encouraging them to go, you know those sort of things. General Practitioner 2*

*Very often there’ll be interruptions in claims, benefits will get suspended. If I could talk to [the Council] at that time when the client’s here I could stop that happening, whereas now… the letters’ll be god knows where… and in the meantime you know the benefit might get suspended… If I could talk to them I could solve a lot of problems because I can put in a nutshell what the client might struggle to sort of want to put across. Staff 3*

Many SP organisations are in the voluntary sector and are isolated from statutory services. Data sharing is therefore problematic. Information is not passed between organisations in a timely manner and even when it is, there is no interoperability in IT systems. The lack of professional status of SP staff leads NHS and other staff to be unsure about what information they can or cannot share with them.

### Mid-range theory

#### Salutogenesis

Central to the model of salutogenesis is the concept of a sense of coherence which is a ‘generalised, long lasting way of viewing the world and one’s place in it’ [27]. Although considered to be stable in adults, it can be altered particularly by radical changes. Additionally, it influences whether or not an individual attempts to change their situation [27]. People with strong sense of coherence, view the world as predictable and therefore comprehensible. Antonovsky links this theory to those of Bowlby (attachment) [31] and Seligman (learned helplessness) [32] while highlighting differences. The sense of coherence is considered to be a continuum from strong to weak, characterised by a normal distribution [27].

Salutogenesis is an individual level explanation of health behaviours. Previous studies have shown a relationship between a strong sense of coherence and good perceived health, particularly mental health [33]. It also seeks to explain why some individuals do not respond to health information from professionals. From a salutogenic perspective, this should not be seen as an individual failure but a failure of the service to provide understandable information [33]. Salutogenesis also refers to the ability to utilise resources (both external and internal) to manage stressful situations. The ability to recognise and use these resources is important for sense of coherence.

Clients reported that staff helped their understanding of their issues. They found health trainers to be motivating and knowing they had someone to turn to helped them to feel that their situation was more manageable. The location of SP away from statutory health services and the ability of staff to work differently to health staff (with difference in time and role flexibility) aided acceptability of the service. However, the fact that it was a non-statutory service did cause problems for information sharing. In this way the client’s comprehension and understanding was often improved but this is not always the case for SP and primary care staff. SP can empower people to utilise their resources and develop new ones. Resources can be internal, such as confidence or self-esteem, or external, such as friends or community who provide advice, support, or bolster internal resources. SP can be considered an external resource but there are many facets to this, due to the different models of SP that exist. However, examples include, the link worker as source of support in a crisis or as a way to access other sources of support, and the community groups provided as part of SP that offer support, companionship and advice.

## Discussion

### Summary

SP in this setting was a not collaborative healthcare intervention but rather a client led, person-centred, asset-based service addressing the wider social determinants of health including co-morbid conditions. In doing so, it worked on salutogenic principles, providing new resistance resources, helping people access existing ones and even strengthening low sense of coherence.

Personalised care is at the heart of social prescribing, staff refer to goal planning in SP as ‘ their plan not mine’; SP goals are set by the clients not staff. Even when clients are referred by health care staff for specific purposes, if that is not what the client wishes to address first (or at all) then this is not a condition of entring the service. Whilst this personalised approach clearly has its merits, and the repeated return to SP organisations and word of mouth recommendations show it is a popular policy, it may disadvantage those whose sense of coherence is so low that they cannot recognise or articulate the need to change. However, given the broad range of activities that is available via SP this should be less of a disadvantage than in traditional health care settings. It also suggests a significant departure from the collaborative framework of collaborative care. The client is not being consulted by the healthcare professional; they are leading the direction of the intervention. This has the potential to have an effect on the level of dependancy people have on the service. However, although many of the interviews with clients mentioned dependecy it was in relation to others. This suggests participants were aware of it but that it might be difficult to recongise or admit to, it is however, an issue that is both live and sensitive.

### Comparison with previous studies

Pelikan concluded that salutogenic thinking has good potential to be applied in health settings [34]. Specifically in health promoting interventions, structures and cultures and improving a person’s sense of coherance could be an explicit goal of chronic disease management [34]. The wider determinants of health must not be underestimated when planning SP delivery [35], they are part of the context to our realist theory and may constrain the effects of the SP intervention. However, this does not invalidate the efforts made in providing these services. For example, people in financial hardship may struggle to access a service that requires bus travel to attend. One of the things participants liked about the organisation studied in this research was that it provided services in several local community locations, meaning most people could walk to services, knew the area and felt comfortable there.

Although sense of coherence is stable, it can be changed, but to do so in a positive way is slow and takes ‘hard work’, such as undertaking psychotherapy [27]. More recent research shows that sense of coherence can change across the life course and that many prerequisites for strengthening sense of coherence may be provided by or mediated by the community [36]. The version of SP that was delivered by the provider organisation, at the time of our research, and the ways that it was experienced by clients, was consistent with the theory of salutogenesis. This research and that of Payne and colleagues [5] found that SP facilitated change in perceptions of personal assets through personal and social development. Meaning that clients became more aware of what assets were available to them and more able to access them. This is consistent with theory of change for the sense of coherence and therefore supports Health Education England’s suggestion that the theoretical base for SP is Salutogenesis [4].

When considering what aspects of SP works for whom and in what circumstances, context is important. Access to a supportive community can strengthen a person’s sense of coherence through the life course [36]. People with a low sense of coherence may struggle to access these resources without help and these people can particularly benefit from SP interventions, although the specific intervention needs to be determined on a case by case basis and remain person centred.

A recent realist review [3] reported that there are three stages which contribute to pathway success in social prescribing. Enrolment, engagement and adherence and the link workers are key to avoiding disruption of the process. Our findings are consistent with these points. Link workers have a key role in ensuring people are supported to attend and understand what social prescribing is. Similarly, another recent realist review of social prescribing had two main concepts, creating and sustaining buy-in, and establishing and maintaining connections [37]. The first of these was not a major concern in our study as referrers were enthusiastic about social prescribing and keen to work with the organisation. Clients too, would regularly report recommending the organisation to friends and neighbours. The caveat here is that both were self-selecting samples and we did not (and could not) gauge how representative they were. The second, maintaining connections, was more of an issue, both in terms of logistics around data sharing and governance, but also regarding remit and scope of the social prescribing provider and its services. These changed over time and some family doctors reported that they were unsure about which service to refer too. This was the reason for developing the triage role, so that referrers had a single point of contact to improve relations. Our findings complement Tierney et al.’s conclusions that social prescribing can, through developing wider social networks, reduce isolation, increase meaning and activity and give people the confidence to manage their own health. Our theory development diverged from that of Tierney et al., whose primary focus was on the setting up of services whereas we studied a mature service that had existed for many years. This allowed us to look beyond logistical considerations into deeper theory behind social prescribing in action.

### Strengths and limitations

SP providers are very varied. The service we studied was provided by a large organisation was large and comprised multiple interventions. As the local authority did not have a central SP referral point the organisation had to liaise with referrers directly, attempting to find local solutions to a city-wide problem. This situation is not the same in other cities or for small single intervention SP groups. However, the central finding of the importance of being client centred and of acknowledging wider determinants of health remain relevant to a wide variety of different SP models of implementation.

This study focussed on an organisation that predominantly served deprived white working-class council estates. There were limited opportunities to capture the voices of people from minority ethnic and non-white British backgrounds.

### Implications for practitioners and commissioners

We sought a better understanding of the mechanisms of action for social prescribing, to provide greater clarity about who can be expected to benefit and why, and therefore who to prioritise in referral practices. Although in-depth, our study was based on a single social prescribing provider in one city and our findings need to be viewed cautiously.

We found that people with long-term social difficulties who struggle with chronic health conditions because they have limited support networks may benefit the most from social prescribing. Our results support the view that this is because building support networks and developing coping mechanisms enables better engagement with sources of treatment. We therefore recommend that these individuals should be considered a priority for social prescribing referrals. It is important to note that those with the weakest sense of coherence, who might be expected to benefit most from social prescribing, may lack sufficient resources to access the service and may need significant help (from referrers and social prescribing providers). A key barrier to this is difficulty in information across organisational boundaries, and those who commission SP services are perhaps best placed to address this.

We found that flexibility on the part of link workers was critical, and they require sufficient time and resources to work this way. Link workers in our study recognised the need to support engagement and that regular attendance was key for reducing isolation. Processes or policies regarding discharge based on time spent in the service were therefore felt by many to be counter-productive, although the consequences for service capacity was also recognised. Commissioners and service providers need to find the correct balance for their chosen aims as different organisations may need to work in different ways to satisfy budgets and local need.

### Implications for research

Theory development moved from a large scale IPT of SP, then modified to the SP organisation in the study then widened out again for the mid-range theory. The mid-range theory therefore should be tested in additional SP organisations to ensure it is not context specific.

## Conclusions

Although superficially similar, social prescribing does not appear to operate as a type of collaborative care. Collaborative care does represent a move towards more holistic thinking within healthcare settings, but social prescribing takes this concept even further. It is holistic and person-centred and as such may enable those with a weak sense of coherence to strengthen this, access resistance resources and move in a health promoting or salutogenic direction.

## Data Availability

Data from this study may be available upon reasonable request to the authors. Individual participant data that underlie the results reported in this article will only be available after deidentification (text, tables, figures and appendices) if the requestor has Research Ethics Committee approval to work with data without gaining additional consent from the original participants. The study protocol will be available. The data will be accessible beginning 3 months and ending 5 years following article publication to researchers who provide a methodologically sound proposal and only to achieve the aims of that proposal. Proposals should be directed to e.f.wood@sheffield.ac.uk. To gain access requestors will need to sign a data access agreement.

## Abbreviations

SP: Social Prescribing
IPT: Initial Programme Theory
CMO: Context-mechanism-outcome
C: Context
M: Mechanism
O: Outcome
